# A Pilot Study of the Synergy between Two Antimicrobial Peptides and Two Common Antibiotics

**DOI:** 10.3390/antibiotics8020060

**Published:** 2019-05-09

**Authors:** Franziska Kampshoff, Mark D. P. Willcox, Debarun Dutta

**Affiliations:** 1 School of Life Science, Engineering & Design, Saxion University of Applied Science, 7513 AB Enschede, The Netherlands; franziska.kampshoff@gmx.de; 2 School of Optometry and Vision Science, University of New South Wales, Sydney, NSW 2052, Australia; duttadebarun@gmail.com; 3 Ophthalmic Research Group, Optometry School, Aston University, Birmingham B4 7ET, UK

**Keywords:** synergy, antimicrobial peptides, antibiotics, *Staphylococcus*, *Pseudomonas*

## Abstract

Background: Frequent and unrestricted use of antibiotics has been associated with the development of antibiotic resistance by microorganisms. Thus, there is a need to find novel antibacterial agents or a combination of agents as the first line of treatment for various infections. This study aimed to investigate the synergy between antimicrobial peptide (AMP) combinations or between AMP-antibiotics combinations using two common pathogens, *Pseudomonas aeruginosa* and *Staphylococcus aureus*. Methods: The AMPs melimine, Mel4 and protamine, and antibiotics cefepime and ciprofloxacin were used in this study. The minimum inhibitory concentration (MIC) of each were evaluated against *P. aeruginosa* and *S. aureus* strains by a microtiter broth dilution. Based on the MIC of each antimicrobial agent, a checkerboard assay was performed to investigate the synergy between them, which was expressed as the fractional inhibitory concentration (FIC). Results: The combination of melimine and ciprofloxacin showed synergistic activity against antibiotic sensitive or resistant strains of *P. aeruginosa* and with FIC values ≤0.5. Conclusion: Combinations of AMPs and the fluoroquinolone ciprofloxacin is a promising method for reducing resistance to the fluoroquinolone of *P. aeruginosa*.

## 1. Introduction

Treatment for infections caused by drug-resistant microorganisms, such as methicillin-resistant *Staphylococcus aureus* (MRSA) and multidrug-resistant *Pseudomonas aeruginosa* is troublesome with current antibacterial therapies. Due to this, there is an increased demand to create new therapies against resistant infections. Combination therapies or alternatives to antibiotics are being investigated as new treatments against drug-resistant bacterial infections [1]. 

Antimicrobial peptides (AMPs) have emerged as promising antimicrobial agents and could be used as alternatives to antibiotics. AMPs have broad-spectrum activity against different gram-positive and gram-negative bacteria, including activity against drug-resistant bacteria [1]. Previous studies have shown active synergism between two AMPs, originally isolated from frogs, Magainin 2 and PGLa [2]. Further possible therapeutic methods include combinational therapies between AMPs and traditional antibiotics. AMPs permeabilize and interact with the membrane of microorganisms in a very different manner than traditional antibiotics [3]. Therefore, AMPs might increase bacterial membrane permeability, providing increased access to the bacterial inner compartment for other antimicrobials [1]. For future treatment options, it is of interest to know whether AMPs could be used in combination with other AMPs or with antibiotics.

We have developed two novel AMPs, melimine and its shorter version Mel4. These have a broad spectrum of activity, being active against gram-positive and gram-negative bacteria (including *Streptococcus pneumoniae, Elizabethkingia meningoseptica, Stenotrophomonas maltophilia, Escherichia coli, Serratia marcescens, Burkholderia cepacia, Delftia acidovorans* as well as multi-drug resistant strains of *Staphylococcus aureus* and *Pseudomonas aeruginosa*), fungi (*Candida albicans, Fusarium solanii*), and the protozoan *Acanthamoeba* [4,5]. Melimine and Mel4 retain activity when coated on a variety of materials including (hydroxyethyl) methacrylate, silicone, titanium, glass, polystyrene, and Teflon [5]. Melimine and Mel4 are also active in animal models and during human clinical trials [6]. One of the parent peptides from which melimine and Mel4 were derived, protamine, has been shown to be active against bacteria and *Acanthamoeba* in combination with the disinfectant polyhexamethylene biguanide [7]. Therefore, the current study aimed to evaluate the synergy between different melimine, Mel4, and protamine combinations, as well as between different AMP–antibiotic combinations against *P. aeruginosa* and *S. aureus* strains.

## 2. Results

### 2.1. Minimum Inhibitory Concentrations

The minimum inhibitory concentration (MIC) of melimine, Mel4, protamine, cefepime, and ciprofloxacin against *P. aeruginosa* and *S. aureus* is shown in Table 1. As strain *P. aeruginosa* 37 was only used in later experiments, its MIC was only determined against ciprofloxacin and melimine.

*P. aeruginosa* 37 was resistant to ciprofloxacin having an MIC of 16 µg/mL, which is 16 times higher than non-resistant *P. aeruginosa* 6294 strain. *P. aeruginosa* 6294 and *S. aureus* 31 were sensitive to cefepime and ciprofloxacin. There are no published breakpoints for Mel4, melimine or protamine, but *P. aeruginosa* 6294 was relatively resistant to protamine.

### 2.2. Synergy between Antimicrobial Agents

**Figure 1:**
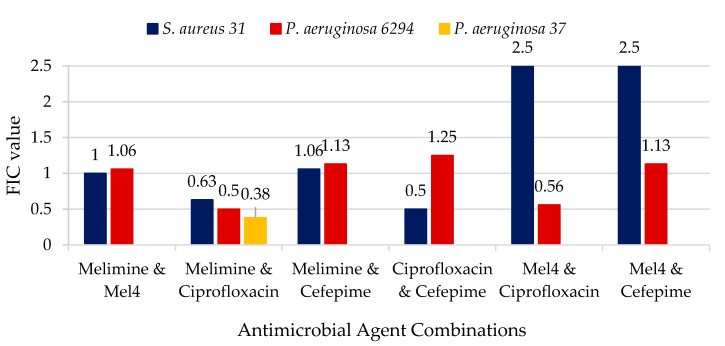
Fractional inhibitory concentrations of the combination of antimicrobial peptides (AMPs) and antibiotics.

For the analysis of the synergy between the different antimicrobial agents, a checkerboard assay was performed. The synergistic activities of the antimicrobial combinations are detailed in Figure 1. Protamine was not synergistic with any other antimicrobial, and so the results are not included in Figure 1. The combination of melimine and ciprofloxacin had synergic effects against *P. aeruginosa* giving fractional inhibitory concentrations (FIC) of ≤0.5. The combination of Mel4 and ciprofloxacin for *P. aeruginosa* 6294 did not reach the FIC needed to be considered synergistic, but it was only just outside the range (FIC = 0.56). There was no synergy between AMPs and the 4th generation cephalosporine cefepime. For *S. auerus* 31 only, there was synergism between cefepime and ciprofloxacin. None of the combinations resulted in antagonism between the antibiotics or AMPs (FIC > 4.0).

### 2.3. Comparison of Synergistic Effects of Melimine with Ciprofloxacin Against P. aeruginosa Strains

**Figure 2:**
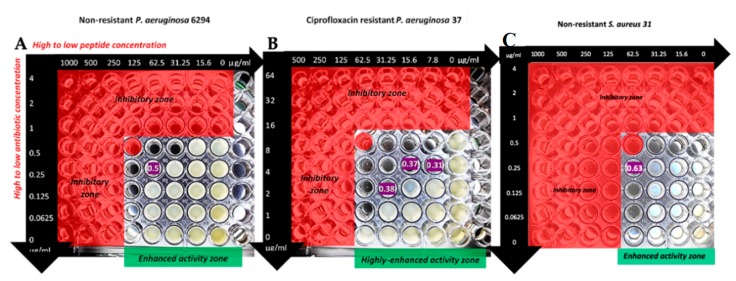
Results of checkerboard assay of the combination of melimine and ciprofloxacin with *P. aeruginosa* 6294 (**A**), *P. aeruginosa* 37 (**B**) and *S. aureus* 31 (**C**). Fractional inhibitory concentration (FICs) values are shown in purple. The red zones demonstrate the inhibitory zones based on the minimum inhibitory concentration (MIC) results.

The synergy between melimine and ciprofloxacin was further analyzed from the checkerboard assay (Figure 2).

For the ciprofloxacin resistant strain *P. aeruginosa* 37, the addition of melimine at 1/8th MIC reduced the MIC by eight times. When the MIC of melimine was reduced to 1/16th or 1/32nd the MIC of ciprofloxacin was reduced by four times on both occasions (Figure 2 wells highlighted as FIC 0.37 and 0.31, respectively). The ratio of melimine and ciprofloxacin used for the synergistic FIC values against *P. aeruginosa* 37 is detailed in the Table 2.

## 3. Discussion

This study demonstrated that the AMP melimine and the traditional antibiotic ciprofloxacin were synergistic with *P. aeruginosa* strains including a multidrug-resistant strain. Furthermore, there was synergy for the *S. aureus* strain studied only between ciprofloxacin and the 4th generation cephalosporin cefepime. No other combinations were synergistic, but none were antagonistic. The AMP melimine is known to interact and destabilize both the outer and inner membranes of *P. aeruginosa* [5], which is characterized by membrane blebbing, loss of membrane integrity, and increased membrane permeability [5]. Ciprofloxacin is a broad-spectrum fluoroquinolone which acts by inhibiting DNA gyrase and topoisomerase types II and IV, [8] both of which are located in the cytoplasm. The membrane destabilization and permeability produced by melimine likely allowed better access for ciprofloxacin. Additionally, intracellular ciprofloxacin can reduce the ability of bacteria to repair their membranes [9], and this may also help the synergy. Resistance to ciprofloxacin, as seen with strain *P. aeruginosa* 37, occurs due to efflux of intracellular ciprofloxacin [10]. Four efflux pumps that are located in the cell membrane are known to facilitate resistance to fluoroquinolone in *P. aeruginosa* [11]. These efflux pumps are energy dependent [12], and so the ability of melimine to depolarize the inner membrane of *P. aeruginosa* [13] would prevent adenosine triphosphate formation and so would likely inhibit the functioning of the efflux pumps. The combination of Mel4 and ciprofloxacin did not reach the cut-off for being considered synergistic, although the FIC was 0.56. Perhaps Mel4 does not cause as much membrane depolarization as melimine. Melimine is longer and contains the membrane-interacting amino acid tryptophan [14,15], which is absent in Mel4 [6]. Furthermore, Mel4 causes a weaker effect on tethered lipid bilayers compared to melamine [16]. The AMP bovine lactoferricin is synergistic with ciprofloxacin with the majority of strains of *P. aeruginosa* isolated from ocular infections [17].

None of the AMPs were synergistic with either antibiotic when tested with *S. aureus*. Similarly, bovine lactoferricin was not synergistic with ciprofloxacin or ceftazidime with *S. aureus* strains from ocular infections [17]. Melimine has a different mode of action against *S. aureus* compared to *P. aeruginosa* [13]. While melimine depolarizes the membrane of both *P. aeruginosa* and *S. aureus*, the depolarization with *S. aureus* does not induce immediate cell death as it does with *P. aeruginosa* [13]. This suggests that other processes are involved in staphylococcal cell death after exposure to melimine and that *S. aureus* may be able to temporarily recover from membrane depolarization which might, therefore, not allow passage of higher concentrations of ciprofloxacin into the cells.

There was a lack of synergy of the AMPs with cefepime with either *P. aeruginosa* or *S. aureus*. Although both these agents act at the cell surface, AMPs act on membranes, whereas cephalosporins act of cell wall synthesis. While there was no synergy, there was also no antagonism (FIC > 4.0) suggesting that both agents work separately. Two other AMPs, DP7 and CLS001, were generally not able to synergize with the β-lactam antibiotic amoxicillin in *S. aureus* or *P. aeruginosa* [18], and two other AMPs, FA1 and Pin2 [G], were not able to synergize with the β-lactams amoxicillin or ceftriaxone in *Klebsiella pneumoniae* [19]. Furthermore, using the strict definition of FIC that is used in the current manuscript, none of a series of 20 AMPs could synergize with carbenicillin in *P. aeruginosa*, but some of the AMPs could synergize with ciprofloxacin [20]. On the other hand, the AMP Esculentin (1–18) does synergize with cephalosporin C [21], and the AMP temporin L can synergize with ampicillin, carbenicillin, and cephalosporin C but not amoxicillin [22]. in *Escherichia coli.* Soren et al. [23] demonstrated that in the majority (60–80%), but not all, *Enterobacteriaceae* tested, the AMP novicidin synergized with ceftriaxone and ceftazidime. It appears that synergy of β-lactam antibiotics with AMPs may be rare in *P. aeruginosa* or *S. aureus* and synergy may also be highly dependent on the AMPs used. Further work is required to more fully understand the synergy of melimine and Mel4 with antibiotics in various different bacterial types.

## 4. Materials and Methods

Melimine (amino acid sequence: TLISWIKNKRKQRPRVSRRRRRRGGRRRR) and Mel4 (amino acid sequence: KNKRKRRRRRRGGRRRR) were synthesized by the conventional solid-phase peptide synthesis protocol and were obtained from Auspep (Tullamarine, Australia). Peptides with >80% purity were used in this study. Melimine is a chimera of the antimicrobial peptides melittin and protamine that we developed to have board spectrum antimicrobial activity. Two different class of antibiotics were used in this study; cefepime is a fourth-generation cephalosporin and ciprofloxacin is a second-generation fluoroquinolone. Ciprofloxacin, protamine and cefepime were procured from Sigma-Aldrich (St Louis, MO, USA). The two antibiotics were chosen as fluoroquinolones, such as ciprofloxacin and cephalosporins, such cefepime as are commonly prescribed to treat *P. aeruginosa* keratitis.

### 4.1. Minimum Inhibitory Concentration (MIC)

*P. aeruginosa* 6294 and *P. aeruginosa* 37 are isolated from corneal infections and *S. aureus* 31 is an isolate from a corneal inflammatory event. *P. aeruginosa* 37 is resistant to ciprofloxacin, gentamicin, levofloxacin, ceftazidime, moxifloxacin, imipenem, ticarcillin, and aztreonam [24]. The two bacterial strains *S. aureus* 31 and *P. aeruginosa* 6294 were tested for their sensitivity to the antibiotics cefepime, and ciprofloxacin, and the AMPs melimine, Mel4, and protamine. *P. aeruginosa* 37 is a drug resistant strain that was tested for its sensitivity to melimine and ciprofloxacin only. The sensitivity was determined by measuring the MIC using the microtiter broth dilution method based on the protocol of the Clinical and Laboratory Standards Institute (CLSI) [25]. Briefly, the bacterial strains were grown in Muller–Hinton broth (MHB; Oxoid, Basingstoke, UK) overnight. Afterward, they were centrifuged for 10 min at 5000× *g*, washed once in phosphate buffered saline (PBS; Na_2_HPO_4_ 1.15 g/L, KH_2_PO_4_ 0.2 g/L, KCl 0.2 g/L, NaCl 8 g/L) and the turbidity of each bacterial solution was diluted in dilution medium up to an optical density (OD) of 0.1 at 660 nm (approximately 10^8^ CFU/mL). These bacterial stock solutions were diluted by 1:500 to a concentration of 2 × 10^5^ CFU/mL, which was confirmed with a retrospective viable plate count. The antimicrobial agents were dissolved in MHB (MHB+; containing 0.2% (*w/v*) bovine serum albumin, 0.01% (*v/v*) acetic acid) and were diluted two-fold for 10 iterations in a 96-well polystyrene microplate (COSTAR, Corning Incorporated, New York, NY, USA). Afterward, the bacterial suspension was added to the wells containing varying antimicrobial agents. Wells with microorganisms but without antimicrobial agents acted as the positive control and the wells with only dilution medium were used as negative controls. The microtiter plates were covered with a sterile lid to prevent evaporation and were incubated at 37 °C for 18 to 24 h with shaking at 120 rpm. After incubation, the 96-well plate was analyzed by visual inspection and the OD measured by a spectrophotometer at 660 nm. The MIC was determined as the lowest concentration that reduced bacterial growth by >90% compared to the well with bacteria alone.

### 4.2. Fractional Inhibition Concentation (FIC)

Synergistic activities between combinations of the antimicrobial agents were tested initially with *P. aeruginosa* 6294 and *S. aureus* 31, and then the activity of the combination of melimine and ciprofloxacin was tested using the drug-resistant strain *P. aeruginosa* 37. Synergy was determined by measuring the FIC using a checkerboard synergy assay. Bacterial strains were grown in Muller–Hinton broth (MHB; Oxoid, Basingstoke, UK) and then resuspended in MHB+ to 2 × 10^5^ CFU/mL. All antimicrobial agents were dissolved in MHB+ at 16 × MIC and then each antimicrobial combination were separately diluted two-fold in MHB+ in both the horizontal and vertical wells of a 96-well polystyrene microplate (COSTAR). The final volume of every well in the final 96-well plate was 100 µL. Following this, the bacterial suspensions were inoculated into every well to give a final volume of 200 µL. Wells without antimicrobial agents but with bacteria acted as the positive control and wells with only medium acted as the negative controls. The microtiter plate was covered with a sterile lid to prevent evaporation and was incubated at 37 °C for 18 to 24 h with shaking at 120 rpm. After incubation, the 96-well plates were visually analyzed for sterility, and the OD was measured by a spectrophotometer at 660 nm. The following formula was used for the calculation of FIC.

$$FIC\;index=FIC\;A+FIC\;B=\;\frac{MIC_{A}^{Combination}\;}{MIC_{A}^{alone}}+\;\frac{MIC_{B}^{Combination}\;}{MIC_{B}^{alone}}\;$$

The FIC was determined as the wells without any visible growth. Synergy occurred at an FIC ≤ 0.5, antagonism at FIC > 4.0 and no interaction occurred at FICs > 0.5 to ≤ 4.0 [26].

## 5. Conclusions

This study showed that the combination of the AMP melimine and ciprofloxacin worked synergistically against two strains *P. aeruginosa*. The smaller AMP Mel4 and ciprofloxacin was not synergistic with *P. aeruginosa,* but the FIC was only slightly higher (FIC = 0.56) than the synergy cut-off (FIC ≤ 0.5). Most other combinations showed no interactions, but none were considered to be antagonistic. Further studies are required to determine the mechanism of synergy between AMPs and antibiotics. 

## Figures and Tables

**Table 1:** Minimum inhibitory concentration (MIC) of the five antimicrobial agents against *S. aureus* 31, *P. aeruginosa* 6294 and PA37.

Bacteria	Antimicrobial Agent				
	Cefepime μg/mL	Ciprofloxacin μg/mL	Mel4 μg/mL	Melimine μg/mL	Protamine μg/mL
*S. aureus* 31	2	1	125	125	250
*P. aeruginosa* 6294	1	1	250	250	1000
*P. aeruginosa* 37	Not done	16	Not done	125	Not done

**Table 2:** Synergistic fractional inhibitory concentration (FIC) values for the combination of melimine and ciprofloxacin against *P. aeruginosa* 37.

Antimicrobials	Inhibitory Concentration (μg/mL)	FIC Value	Inhibitory Concentration (μg/mL)	FIC Value	Inhibitory Concentration (μg/mL)	FIC Value
melimine	7.8	0.31	15.6	0.37	31.25	0.38
ciprofloxacin	4		4		2	
